# Relevant and Non-Redundant Feature Selection for Cancer Classification and Subtype Detection

**DOI:** 10.3390/cancers13174297

**Published:** 2021-08-26

**Authors:** Pratip Rana, Phuc Thai, Thang Dinh, Preetam Ghosh

**Affiliations:** Department of Computer Science, Virginia Commonwealth University, Richmond, VA 23284, USA; ranap@vcu.edu (P.R.); thaipd@vcu.edu (P.T.); tndinh@vcu.edu (T.D.)

**Keywords:** feature subset selection, disease classification, subtype detection

## Abstract

**Simple Summary:**

Here we introduce a new feature selection algorithm DTA, which selects important, non-redundant, and relevant features from diverse omics data. DTA selects non-redundant features by maximizing the similarity between each patient pair by an approximate k-cover algorithm. We successfully applied this algorithm to three different biological problems: (a) disease to healthy sample classification, (b) multiclass classification of different disease samples, and (c) disease subtypes detection. DTA outperformed other feature selection techniques in the binary classification of healthy and disease samples and multiclass classification of various diseases. It also improved the performance of a subtype detection algorithm by selecting the important features for few cancer types.

**Abstract:**

Biologists seek to identify a small number of significant features that are important, non-redundant, and relevant from diverse omics data. For example, statistical methods such as LIMMA and DEseq distinguish differentially expressed genes between a case and control group from the transcript profile. Researchers also apply various column subset selection algorithms on genomics datasets for a similar purpose. Unfortunately, genes selected by such statistical or machine learning methods are often highly co-regulated, making their performance inconsistent. Here, we introduce a novel feature selection algorithm that selects highly disease-related and non-redundant features from a diverse set of omics datasets. We successfully applied this algorithm to three different biological problems: (a) disease-to-normal sample classification; (b) multiclass classification of different disease samples; and (c) disease subtypes detection. Considering the classification of ROC-AUC, false-positive, and false-negative rates, our algorithm outperformed other gene selection and differential expression (DE) methods for all six types of cancer datasets from TCGA considered here for binary and multiclass classification problems. Moreover, genes picked by our algorithm improved the disease subtyping accuracy for four different cancer types over state-of-the-art methods. Hence, we posit that our proposed feature reduction method can support the community to solve various problems, including the selection of disease-specific biomarkers, precision medicine design, and disease sub-type detection.

## 1. Introduction

Omics data usually comprise thousands of features; however, most of these features are redundant, irrelevant, or noisy. Experimental noise, multiple intrinsic interconnections between the biological units, and co-regulation between the features are possible reasons for redundancy. For example, typical RNA-seq measurements catalog the expression of thousands of transcripts; however, most of them are redundant (i.e., highly correlated) or noisy. Moreover, due to the experimental costs, the number of samples available is lower than the number of features, making the traditional machine learning and statistical algorithms easily overfit the biological data. Another problem is the lack of control/normal samples; this is mainly because there are fewer chances to collect data from healthy patients. Therefore, selecting a small number of relevant and non-redundant features among the complete set of features is a significant research problem.

Yu et al. classified the genes in a disease into the following four categories: (a) irrelevant or noisy genes; (b) weakly relevant and redundant genes; (c) weakly relevant and non-redundant genes; and (d) strongly relevant genes [1]. Generally, researchers wish to select a small number of genes that are either strongly relevant or weakly relevant and non-redundant. The selection of this subset of relevant genes is essential for several biological problems, such as identifying causal disease-related genes, the early detection of diseases, designing precision medicine, and disease sub-type detection [2]. In machine learning, a similar problem is termed the feature selection problem, where the goal is to select the most informative and small subset of features from a larger number of features. Feature selection becomes critical when only a few samples are available compared to the number of features in the dataset; it reduces noise, improves the training time of the machine learning models, and prevents over-fitting. Ang et al. [2] classified the feature selection techniques into the following five categories: (a) filter; (b) wrapper; (c) embedded; (d) hybrid; and (e) ensemble. Several of these feature selection techniques have been used in the past for different purposes. For example, *twoPhase* [3]; *iterFS* [4]; *WeiBi* [5] are based on information gain, LASSO, or Fisher score. Feature selection algorithms in the biological domain were briefly discussed in [2,6].

In this paper, we propose a new feature selection method and demonstrate its performance in (i) classifying disease samples from normal samples; (ii) classifying the different types of disease samples; and also (iii) disease subtype detection. First, we evaluated the performance of our method in disease classification considering six different large cancer gene expression datasets from The Cancer Genome Atlas (TCGA) [7]. Then, we benchmarked our results using three different feature/geneset selection methods and a few differential expression (DE) methods on the same dataset. From the results, we found that our proposed method outperformed all the popular geneset selection methods and can identify essential genes and functional pathways in diseases in general and particularly cancer.

## 2. Materials and Methods

### 2.1. Method Overview

**Figure 1:**
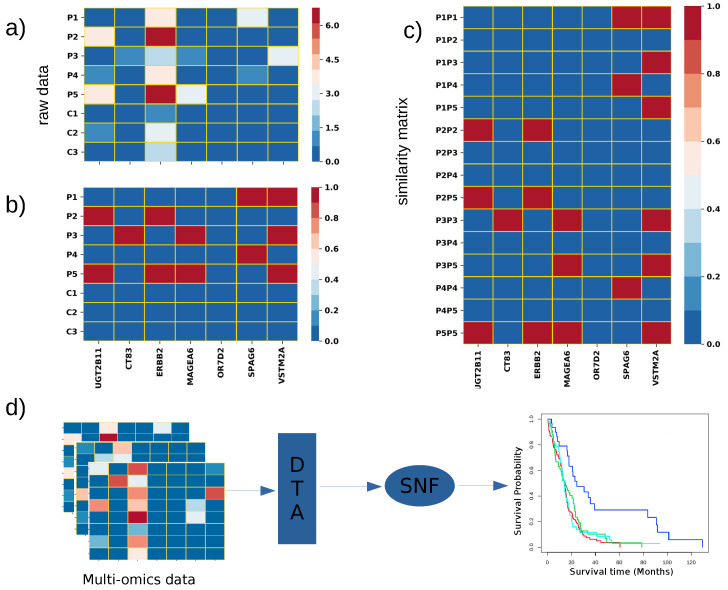
Overview of the method: (**a**) gene expression profiles of the patients; here, Pi denotes the disease sample, and Ci denotes the control sample; (**b**) construction of PEEP matrix from (**a**); (**c**) creating a patient-to-patient (denoted by PiPj) similarity matrix and selecting the column using an approximate k-cover algorithm; (**d**) an overview of the subtype detection pipeline.

Our proposed feature selection method consists of two main steps. First, it creates a binary patient-specific perturbation profile (PEEP) from the genomics dataset using data normalization and imposes a cut-off. Second, it selects non-redundant features that maximize the similarity between each patient pair by an approximate *k*-cover algorithm. The *k*-cover problem in a graph G=(V,E) is an NP-complete problem, which seeks a set of size *k* nodes that cover the maximum number of edges. A standard greedy algorithm can approximate this problem with (1 − 1/e) approximation. However, due to the large space consumption and time complexity which can be a bottleneck for a large graph, several algorithms have been proposed for approximate *k*-cover. We used the dynamic threshold algorithm (DTA) [8] to find the subset *C* of *k* genes that guarantee a $1-\frac{1}{e}-\epsilon$ approximation solution to the k-cover problem. Figure 1 shows a brief overview of the DTA algorithm.

### 2.2. Creating Personalized Perturbation Profile (PEEP)

First, we converted the raw gene expression dataset into a log scale. Consider a set of *n* patients $X=\{X_{1},X_{2},\cdots ,X_{n}\}$, where a patient, $X_{i}$, is represented by *m* features. First, we compute the mean and standard deviation of each feature from the control sample. Then, we perform a column-wise normalization using the following formula:

(1) $$z_{ij}=\frac{x_{ij}-\mu_{j}^{N}}{\sigma_{j}^{N}}$$

Here, $x_{ij}$ refers to the log-transformed raw expression of patient *i* and feature *j*, $z_{ij}$ is the Z-score transformed value of the expression of patient *i* and feature *j*. $\mu_{j}^{N}$ and $\sigma_{j}^{N}$ are the mean and standard deviation of feature *j* in the control samples.

In the next step, we convert the *Z* matrix to the binary ‘0’/‘1’ PEEP matrix $P=p_{ij}$ by imposing a threshold of $z_{t}$. For example, if the absolute value of a feature in a subject has an expression that is greater than $z_{t}$, we consider that as ‘1’, otherwise ‘0’:

(2) $$p_{ij}=\left\{\begin{array}{cc} 0\; & \mathrm{if}\;-z_{t}<z_{ij}<z_{t}\\ 1\; & \mathrm{if}\;-z_{t}>z_{ij}\;\mathrm{or}\;z_{t}<z_{ij} \end{array}\right.$$

Thus, the features having ‘1’ can be viewed as over-expressed/under-expressed in one subject. Similar normalization was performed as before, and we used the same $z_{t}$ cutoff of 2.5 for our analysis [9]. We also performed the same analysis using two other cutoffs (2 and 3) and observed similar results (shown in Supplementary Figure S1).

### 2.3. Feature-Selection Problem Formulation

In our method, we only selected a subset of features to run the classification. Here, we selected *k* features so that we can preserve the maximum information of the patients. By performing gene-selection, we reduced the overfitting of the classification, thereby improving accuracy.

Similarly to the feature-selection problem [10], we considered the dataset as the set of pairs of patients. We defined that two patients are similar if there exists a feature that is up-regulated/ down-regulated in both patients. We build a similarity matrix $S=s_{ij}$ as

$$s_{ij}=\left\{\begin{array}{cc} 1\; & \mathrm{if}\;\exists h:p_{ih}=p_{jh}=1\\ 0\; & \mathrm{if}\;\mathrm{otherwise} \end{array}\right.$$

The goal is to select a subset *C* of *k* features so that we can preserve the similarity matrix. More concretely, we want to maximize the similarity between patients on the subset *C* as follows:

$$C=arg\max_{C\subset G,|C|=k}\sum s_{ij}^{C}$$

where:

$$s_{ij}^{C}=\left\{\begin{array}{cc} 1\; & \mathrm{if}\;\exists h\in C:p_{ih}=p_{jh}=1\\ 0\; & \mathrm{if}\;\mathrm{otherwise} \end{array}\right.$$

### 2.4. DTA Algorithm

The feature-selection problem can be considered as a *k*-cover problem. Let us say a feature *h* covers a pair of patients $\left(X_{i},X_{j}\right)$ if $p_{ih}=p_{jh}=1$; we use the DTA algorithm [8] to find the subset *C* of *k* genes that guarantee a $1-\frac{1}{e}-\epsilon$ approximation solution. However, note that our proposed framework can work seamlessly with other existing algorithms for the *k*-cover problem.

In the DTA algorithm, we sample random hyperedges (each hyperedge consists of a feature and a pair of patients covered by that feature) to select a subset of *k* features.

In particular, we iterate *k* times to select the subset of *k* features. At a specific iteration, we select a feature as follows:

- Add new hyperedges by repeating the following steps.
  - Randomly choose two patients $X_{i}$, $X_{j}$;
  - For each feature h∈G, if $p_{ih}=p_{jh}=1$, add (h,(i,j)) to the hyperedge.
- Select a feature $h^{*}$ that covers the most pairs of patients. For each pair of patients (i,j) that is covered by $h^{*}$, remove all hyperedges that consist of (i,j).

The DTA algorithm has a run-time of $O(\epsilon^{-2}km\log m)$ (where *m* is the total number of genes, and *k* is the number of selected genes) and can quickly scale to thousands of patients’ data.

### 2.5. Classification Workflow

We performed five-fold cross-validation (5-CV) with an 80–20 split on the raw dataset. For each split of the training dataset, we first estimated the mean (μ) and standard deviation (σ) from the healthy samples. We then selected a set of genes from the training set using our method. Finally, we trained the model with a linear-SVM on the selected genes and classified the samples as healthy or diseased samples. Unfortunately, the TCGA dataset is imbalanced as it has fewer healthy samples than disease samples. Hence, we use the ROC-AUC score, false-positive, and false-negative rates to evaluate the classification performance.

We also benchmarked our results with two other feature selection methods, *twoPhase* [3], *iterFS* [4]; one geneset selection method *Barabasi* [9]; and one DE analysis method, *LIMMA*. For twoPhase, iterFS, and Barabasi we follow the same preprocessing as our method to select the subset of features. For *LIMMA*, we directly used raw data as the input as LIMMA takes continuous inputs.

For multiclass classification, we merged the PEEP transformed patient subjects’ data of six cancer types. Furthermore, we performed a 5-CV with an 80–20 split. For the performance evaluation, we first measured the ROC-AUC score of one-to-all classification of one cancer type to all other cancer types. Then, we averaged the ROC-AUC score of one-to-all classifications of the cancer types. Finally, for the multiclass classification, we only benchmarked twoPhase and iterFS, as Barabasi and LIMMA were not designed for the multiclass problem.

### 2.6. Disease Subtyping Workflow

A heterogeneous disease such as cancer is activated through several pathways and shows a high level of molecular heterogeneity. Therefore, causal oncogenes only express in a subset of patients for a cancer type. For example, in the TCGA-lung squamous cell carcinoma (LUSC) dataset, the popular linear DE method *LIMMA* identified approximately 13,852 genes as differentially expressed (p≤0.05) [11]. Interestingly, most of these differentially expressed genes are only perturbed in a few patients. For a heterogeneous disease such as cancer, different perturbations in multiple oncogenes lead to a common phenotypic outcome. The outcome of patients with the same cancer type also significantly differs based on the phenotypic outcome. Thus, disease subtyping is a crucial method to predict disease variability, identify associated molecular pathways, and design a personalized treatment plan for a heterogeneous disease.

The disease subtype prediction from omics data mainly consists of the following two steps. First, it computes patient-to-patient similarity (e.g., Euclidean distance, Pearson correlation) from the omics data. It then performs an unsupervised clustering (e.g., k-means, consensus) on that similarity matrix to group similar patients for identifying the sub-types. Subtype detection is complex and often requires multi-omics data integration of the same patients to achieve better clustering/subtyping. Some of the popular multi-omics data integration methods are iCluster [12], similarity network fusion (SNF) [13], PINS [14], CIMLR [15] and autoencoders [16]. However, these methods often depend on selecting important features from diverse high-dimensional omics datasets (e.g., gene expression, methylation, copy number, miRNA expression). Our proposed DTA method can identify such important features from these multiple datasets and improve the existing sub-type detection methods.

We analyzed the performance of DTA as a feature selection method in the standard subtype detection pipeline using the data integration method SNF. SNF is a network fusion method that first generates a patient-to-patient similarity network from all datatypes individually using a non-linear kernel function. Then, it fuses all individual networks into a single comprehensive network using an iterative cross-network diffusion algorithm. Here, we use gene expression, miRNAs expression, and DNA methylation profiles from TCGA [7] of the same patients to perform subtype detection. Then, we used our feature selection method on the multiview data to individually select essential features from each data type. We created the PEEP profile of each data type separately and ran k-cover to identify the important features. Due to the lack of common healthy samples, the cancer samples themselves were used as control samples. Based on this, we calculated the Euclidean distance (i.e., similarity) for every patient pair. Then, we integrated these three distance matrices into a single comprehensive dataset using SNF. Later, we performed spectral clustering on this Euclidean distance to group similar patients and validate disease subtypes using the Kaplan–Meier curve of the survival rate of the patients.

We used the R tool CancerSubtype for subtyping a cancer type [17]. For feature selection, we used DTA and the other two feature selection methods based on principal component analysis (PCA) and variance (VAR) for comparison. For further analysis, we used the linear model LIMMA to identify DE genes [11], and the ClusterProfiler package for the functional analysis of the DE geneset [18].

## 3. Results

### 3.1. TCGA Dataset

The Cancer Genome Atlas (TCGA) program integrates various molecular profile information of more than 33,000 samples across 68 different cancer types [7]. We performed our classification analysis for RNA-seq data for six different types of cancer from TCGA: breast invasive carcinoma (BRCA); lung adenocarcinoma (LUAD); lung squamous cell carcinoma (LUSC); prostate adenocarcinoma (PRAD); colon adenocarcinoma (COAD); and kidney chromophobe (KICH). We discarded other cancer types in TCGA from our analysis, as they had fewer control samples. We used the TCGA dataset of four cancer types BRCA, COAD, LUSC, and GBM (glioblastoma multiforme), for subtype detection. Three different data types—gene expression, DNA methylation, and miRNA expression—of the same patient set were used herein. To perform the survival analysis, we also downloaded the clinical data of the patients from the TCGA. We used the TCGAbiolink package to load data from TCGA [19].

### 3.2. Classifying Disease Samples with Normal Samples

**Figure 2:**
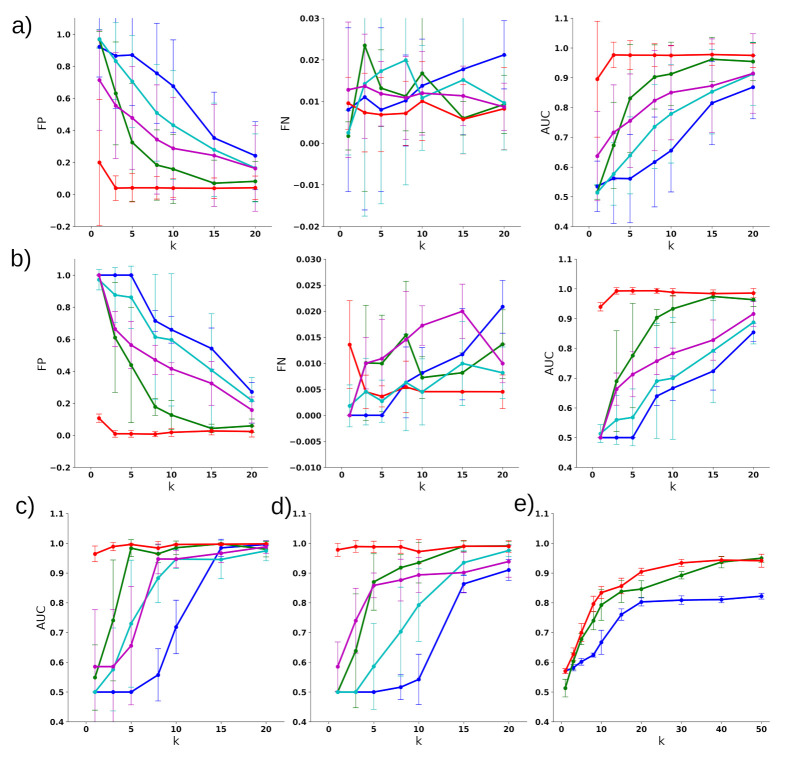
Performance benchmarks of DTA compared to other feature selection methods for disease classification. The blue color denotes PCA, green denotes two-phase, cyan denotes Barabasi, magenta denotes LIMMA, while red denotes DTA geneset: (**a**) the average false-positive, false-Negative, and ROC-AUC score of the SVM classifier over six different disease datasets from TCGA; (**b**) false-positive (FP), false-Negative (FN), and ROC-AUC score for the TCGA-BRCA dataset; (**c**) ROC-AUC score of TCGA-LUSC dataset; (**d**) ROC-AUC score of TCGA-LUAD dataset; and (**e**) ROC-AUC score of multiclass classification for six cancer types from TCGA.

First, we evaluated the performance of DTA for cancer with normal sample classification using gene expression data. We selected a comparatively small *k* (1≤k≤20) number of genes using DTA for each cancer type. DTA performed remarkably well in classifying the diseases of the normal samples compared to the baseline (classification without any feature reduction). Additionally, its performance was quite consistent over different disease types in achieving a high ROC-AUC score with a low FN rate, as shown in Figure 2. We also benchmarked four other methods *twoPhase* [3]; *iterFS* [4]; *Barabasi* [9]; and *LIMMA*. We found that the performance of these algorithms was inconsistent, and that the performance degraded—especially when a small number of features were selected (Figure 2). This shows that these standard feature selection algorithms cannot find the most non-redundant and relevant features that reduce the classification accuracy. The average of the FP, FN, and ROC-AUC scores for the classification of six different cancer types are shown in Figure 2a. DTA achieved an almost perfect ROC-AUC using only three genes, while the other methods struggled to achieve a ∼0.7 ROC-AUC for the same number of selected genes. The FP, FN, and ROC-AUC score of BRCA classification are shown in Figure 2b, and the ROC-AUC of LUSC, LUAD, is shown in Figure 2c,d.

### 3.3. Multiclass Disease Classification

Then, we extended our analysis to evaluate the performance of DTA in a multiclass disease classification problem. We trained a multiclass classification model using SVM with a linear kernel and also performed 5-CV. DTA showed a remarkable improvement in ROC-AUC classification compared to the other gene selection methods and the original data. In particular, DTA achieved approximately 0.9 ROC-AUC for the selected 20 genes. Especially for a small number of selected genes, the performance of the other methods was quite poor. The comparison of ROC-AUC of DTA to the other two feature section methods *twoPhase* and *iterFS* is shown in Figure 2e. These feature selection algorithms performed poorly for a small number of genes compared to DTA.

### 3.4. Disease Subtype Detection

**Figure 3:**
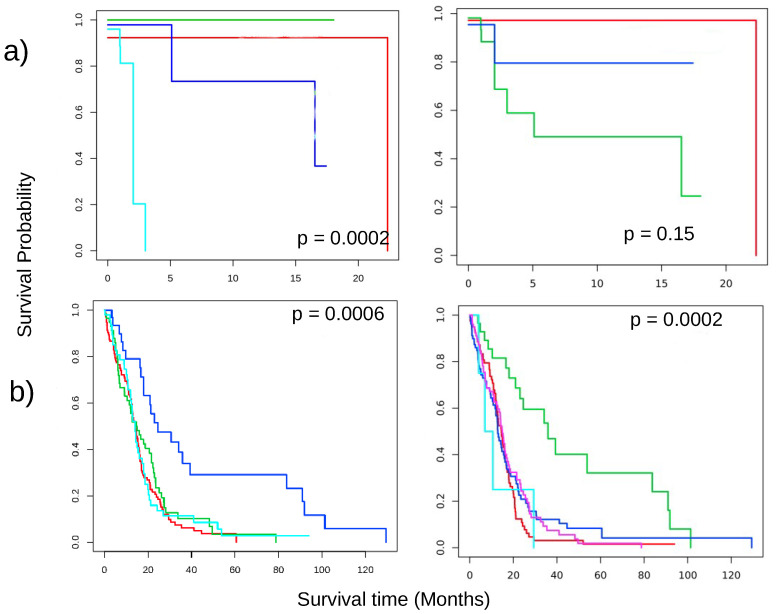
Disease subtype detection using DTA as a feature reduction step. Kaplan–Meier curve of the detected subtypes using DTA (left) and maximum variance (right) selected genes of (**a**) COAD cancer; and (**b**) GBM cancer. The colors indicate the different clusters that were identified. *p*-value signifies that the patients in different subtypes have different survival profiles.

DTA-selected genes performed quite well to identify different subtypes in a cancer type. For most of the cancer types, the identified clusters have a different survival profile. The DTA method improved the *p*-value of the survival profile/Kaplan–Meier curve of the clusters over the baseline (without feature selection) shown in Figure 3. Here, a low *p*-value confirmed that the survival profile of patients in different clusters is significantly different. Furthermore, we benchmarked the result with the other two feature selection techniques: PCA and maximum variance (VAR). DTA showed a better and more consistent performance in most cancer types in terms of the *p*-value of the Kaplan–Meier curve and average silhouette score of the clusters. We also performed a functional analysis on the DE gene set of the identified clusters. This further confirmed that the identified clusters are related to very different biological pathways.

### 3.5. Key Findings on Brca

Breast cancer is the most commonly diagnosed cancer among US women after skin cancer. In the TCGA-BRCA dataset, the DE method, LIMMA, identified 13,493 genes as differentially expressed among 18,934 genes (adj *p*-value < 0.05), most of which were, however, redundant and co-regulated. To identify the non-redundant genes, we applied the DTA method on this TCGA dataset. DTA achieved an almost perfect ROC-AUC only using three genes—while the other methods struggled to achieve 0.7 ROC-AUC using the same number of genes. The genes selected by DTA for k=10 are *ANO3*, *CT83*, *ERBB2*, *MAGEA6*, *OR7D2*, *SPAG6*, *TDRD12*, *TDRD9*, *UGT2B11*, *VSTM2A*. However, surprisingly among these top 10 genes, *SPAG6*, *OR7D2*, *TDRD9*, and *TDRD12* genes were not found to be differentially expressed although the involvement of some of these genes was already mentioned in the literature. For example, *ERBB2* is an oncogene whose involvement has been reported in several studies for the past 30 years [20]. The expression of *MAGEA6* was found to be associated with the poor survival of breast cancer patients [21]. Thus, DTA could find important features (i.e., genes) from the dataset even though their expression profiles did not exhibit significant differential expression. Although DTA picked genes TDRD9 and TDRD12, which belong to the same family, we found their expression pattern to be quite different for cancer samples. We calculated the logical XOR of the PEEP profile for the genes TDRD9 and TDRD12 and found that approximately 6.2% of patients showed a reverse expression profile (i.e., TDRD9 is expressed while TDRD12 is not expressed or vice versa). This value is higher than the expected random value of 4.875% (considering genes follow a Gaussian distribution and a $Z_{t}$ cutoff of 2.5); this suggests that TDRD9 and TDRD12 expression is more different than an average/random gene in this dataset.

We observed that the maximum number of DTA identified genes are differentially expressed. For example, for BRCA cancers, 13,493 genes were found to be differentially expressed (adj *p*-value <0.05). *SPAG6*, *TDRD9*, *TDRD12*, *OR7D2* are the four genes which come in the top ten DTA genes, but are not differentially expressed. Five out of the top ten DTA genes also come under the Barabasi combinatorial pool of geneset: *MAGEA6*, *CT83*, *ERBB2*, *ANO3*, *OR7D2*. Only four genes chosen by DTA are differentially expressed and also present in the Barabasi geneset. These four genes are *MAGEA6*, *CT83*, *ANO3*, *ERBB2*. The top ten DTA genes which are not differentially expressed or come under the Barabasi geneset are *SPAG6*, *TDRD9*, *TDRD12*.

Subtype detection using DTA identified four subtypes of BRCA of 299 patients. The DTA identified subtypes did not show different survival profiles (*p*-value = 0.07). The other feature selection methods also failed to achieve the subtype with significant difference in survival profiles. Subtype 1 has 121 patients, whereas a total of 91 and 87 patients were identified as subtype 2 and subtype 3, respectively, in BRCA. *COL10A1*, *MMP11*, *DMD*, *C10orf90*, and *CNTNAP3* are the top five differentially expressed genes (based on the lowest *p*-value) of BRCA subtype 1. Similarly, the genes *COL10A1*, *MMP11*, *CXCL2*, *CA4*, and *LRRC3B* were the top five differentially expressed genes in subtype 2. The top five differentially expressed genes of subtype 3 are *TPX2*, *KIF4A*, *NEK2*, *CDCA5*, and *COL10A1*.

We compared our approach with the PAM50 enriched BRCA subtype identified by Berger et al. [22] as shown in Table 1. Our subtype 2 mainly includes patients from the Basal subtype which are expected to lose the function of *TP53*, *RB1* and *BRCA1* genes [23]. Fifty-two out of 55 patients in the Basal subtype come under subtype 3. The DTA identified that first subtype also overrepresents the LumA subtype. Note that PAM50 identifies subtypes based on RNA expression only, whereas our method integrates multiview data containing RNA expression, DNA methylation and miRNA expression.

### 3.6. Key Findings on COAD

Colorectal cancer is the fourth-ranked in terms of cancer-related deaths globally. DTA selected the following genes as important indicators of colorectal cancer: *ABCA12*, *CLDN18*, *MAGEA11*, *MAGEA6*, *MTRNR2L1*, *MTTP*, *NPC1L1*, *PRSS21*, *SLC14A1*, and *SPESP1*. *CLDN18* encodes the gastric type adhesion molecule and is a known biomarker for gastric cancer [24]. *MUC5AC* expression is associated with tumorigenesis in colorectal cancer via a serrated neoplasia pathway [25]. Another study found that *MUC5AC* was expressed in pancreatic ductal and various gastrointestinal tract tumors [26]. Colorectal cancer is strongly associated with lipid metabolism; *NPC1L1* is a sterol transporter that is a key regulator of lipid homeostasis. *NPC1L1* knockout mice were found to have a reduced number of tumors than the wild-type mice [27]. *SLC14A1* was identified in intestinal stem cell signature, which is associated with the poor survival of COAD patients [28]. Thus, the genes detected by DTA are significant predictors of colorectal cancer.

DTA also identified four different clusters in COAD by using only 10 features from each of the three data types. These clusters showed an entirely different survival profile (*p*-value < 0.00022). The clusters consisted of 26, 37, 48, and 25 patients, respectively. The top five DE genes of the first cluster are *CDH3*, *CA7*, *PHLPPL*, *GCNT2*, and *ENPP6*. The most related functional pathways considering these genes are cell division, the G2/M transition of the mitotic cell cycle, mitotic nuclear division, regulation of protein serine/threonine kinase activity, leukocyte migration. Top differentially expressed genes in the second cluster are *MYOC*, *ABHD7*, *ABCA8*, *SLC30A10*, and *CDH3*, and the corresponding enriched biological processes are cell division, the G2/M transition of the mitotic cell cycle, cell cycle G2/M phase transition, ncRNA processing, and the ncRNA metabolic process. *CA7*, *CDH3*, *CLEC3B*, *CLDN8*, *SLC30A10* are the most differentially expressed genes in cluster 3, corresponding to the enriched biological processes: mitotic cell cycle phase transition, cell cycle G2/M phase transition, cell cycle phase transition, the G2/M transition of the mitotic cell cycle, and mitotic nuclear division. In the last cluster, the most expressed genes are *ABCA8*, *SFRP1*, *CDH3*, *GCNT2*, *KIAA1199*, and the corresponding enriched GO terms are the regulation of leukocyte migration, leukocyte migration, cell division, mitotic nuclear division, and cell cycle G2/M phase transition.

### 3.7. Key Findings on GBM

Glioblastoma multiforme is the most common malignant brain tumor in adults. DTA selected the following genes in this cancer: *ARHGEF2*, *FRS3*, *IRX2*, *VAT1L*, *NDRG1*, *PDPK1*, *PTK6*, *RAB3C*, *RPS4Y1*, and *WDR18*. *NDRG1* is a tumor suppressor gene with the ability of metastasis and migration of cancer cells. A study found *NDRG1* to be associated with the hypoxia-associated molecule and is expressed in the GBM cell [29]. GBM is also the most studied dataset for subtype detection. Here, we identified four subtypes for 276 GBM patients. The first cluster consists of 137 patients, and the most expressed genes are *IL12RB2*, *CACNB1*, *ICAM5*, *BTN3A2*, and *INPP5F*. The regulation of vesicle-mediated transport, axodendritic transport, neuron death, mitotic cell cycle phase transition, and cell cycle phase transition are the most enriched processes in this cluster. The second cluster consists of 59 patients, and the signature DE genes are *PI4KA*, *IL12RB2*, *CACNB1*, *MICAL2*, and *SLC17A7*. The third cluster has 33 patients with a high survival rate, and the top DE genes are *WSCD2*, *CACNB1*, *KDELR2*, *MICAL2*, and *RYR2*. Mitotic cell cycle phase transition, cell cycle phase transition, axodendritic transport, axonal transport, and regulation of mitotic cell cycle phase transition are the top enriched processes in this cluster. The fourth cluster consists of 47 patients with signature DE genes being *HRH3*, *TSPYL1*, *MAP2K1*, *GOT2*, and *FUT1*. The functional analysis of identified clusters can be found in Supplementary Figures S2–S5.

### 3.8. Key Findings on LUAD

TCGA-LUSC has a dataset of 59 control samples and 533 LUAD patient samples. Here, we achieved almost perfect AUC using only 3 genes using DTA. The top 10 genes selected using DTA are *ABCC2*, *CHRNA9*, *CSAG1*, *EPS8L3*, *INSL4*, *SLC13A2*, *SPESP1*, *TDRD1*, *ZFR2*, and *ZNF560*. One study identified *ABCC2* as an important gene candidate for LUAD using the expression and network analysis [30]. *CSAG1* encodes cancer-germline antigens (CGAs) [31]. Aberrant *INSL4* signaling is related to LKB1-inactivated lung cancer [32]. *CHRNA9* is a Nicotinic Receptor and is related to smoking-induced tumor formation [33]. We did not perform subtype detection for LUAD due to the lack of common patients in the three datatypes used in our analyses.

## 4. Discussion

**Figure 4:**
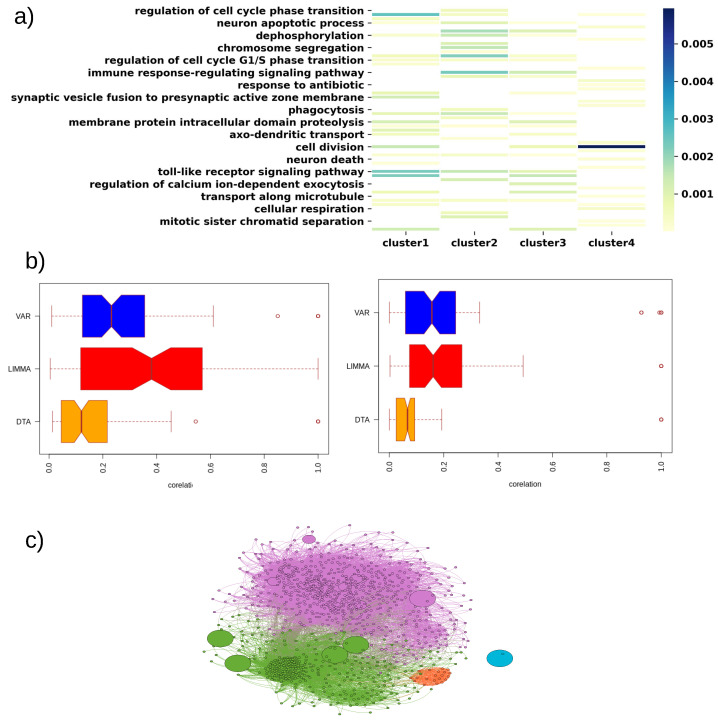
(**a**) Enriched biological functions of each subtype of GBM cancer type where DTA was used as a feature reduction technique. xlabel denotes the enriched biological terms and color intensity represents the *p*-value of the association; (**b**) analysis of how DTA selected features are different from those identified by other feature selection methods. Here, we compared the correction among the genes for different feature selection and DE methods. The left-side figure is for BRCA and the right side is for COAD; and (**c**) Predicted gene–gene interaction network of BRCA. The colors indicate five different clusters that were identified. The big circle shows the DTA selected genes, which are distributed over the network. The medium-sized circle denotes the genes selected by LIMMA, which mainly belong to one cluster (cluster with violet color).

Feature selection is a crucial step of biological data analysis as biological measurements contain a high number of features compared to samples. Here, we present an algorithm that showed a remarkable improvement over existing feature selection techniques for disease classification and subtype detection problems. Genes selected by our algorithm were previously validated as shown in Table 2. Furthermore, we performed a few tests to analyze some of the algorithm’s properties, making it an excellent feature selection method. To ensure each identified cluster in a cancer type is functionally different, we enriched the functional GO terms from the DE genes of each cluster of GBM and COAD cancers. The enriched functions of each cluster are quite different from each other (Figure 4a and Supplementary Figure S6). We also computed the correlation between the selected genes by our algorithm and compared them with the other feature selection methods. We observed a very low correlation between the selected genes, which ensures the capability to choose non-redundant features by our method. The mean gene-to-gene correlation is higher for LIMMA and VAR than DTA, as shown in Figure 4b. Lastly, we predicted the gene regulatory network from the gene expression data of a cancer type. We used a consensus of six different gene-regulatory network prediction algorithms to obtain a high confidence regulatory network based on our previously developed pipeline [34]. Then, we performed a clustering to group similar genes in the network. Thus, the genes belonging to a cluster regulate each other more than regulating genes from the other clusters. We found that the genes selected by DTA belonged to different clusters, as shown in Figure 4c, whereas feature selection methods such as LIMMA and VAR choose the features, i.e., genes that belong to the same cluster.

## 5. Conclusions

In this work, we introduced a novel feature selection technique that selects important and non-redundant disease-related features. We applied DTA for three different biological problems. DTA outperformed other feature selection techniques in the binary classification of healthy and cancer samples and the multiclass classification of various cancers. It also improved the performance of a subtype detection algorithm by selecting the important features for few cancer types. Currently, DTA operates on a patient-specific binary perturbation matrix. Hence, some information can be lost due to discretization to create a patient-specific binary profile. Thus, one potential improvement over our current method is to design extensions of the DTA algorithm to work with the continuous inputs. Theoretically, this can reduce the information loss due to discretization and further improve the results. Currently, our algorithm selects genes purely based on the expression data. Adding the prior knowledge of gene families and their functionality can further improve our feature selection process.

## Figures and Tables

**Table 1:** A comparison of our approach with the PAM50 enriched BRCA subtype identified by Berger et al. [22].

Group	Basal	Her2	LumA	LumB	Normal	Sum
1	1	12	80	26	2	121
2	2	13	44	29	3	91
3	52	10	16	8	1	87
Sum	55	35	140	63	6	299

**Table 2:** List of previously validated genes selected by our algorithm.

Disease	Genes	Comments
BRCA	ERBB2	Involvement is reported in several studies for the past 30 years [20].
	MAGEA6	Associated with poor survival of breast cancer patients [21].
COAD	CLDN18	Encodes gastric type adhesion molecule and known biomarker for gastric cancer [24].
	MUC5AC	Associated with tumorigenesis in colorectal cancer via a serrated neoplasia pathway [25].
	NPC1L1	Key regulator of lipid homeostasis [27].
	SLC14A1	Associated with poor survival of COAD patients [28].
GBM	NDRG1	Associated with the hypoxia-associated molecule and is expressed in GBM cell [29].
LUAD	ABCC2	Important gene candidate for LUAD [30].
	CSAG1	Encodes cancer-germline antigens (CGAs) [31].
	INSL4	Related to LKB1-inactivated lung cancer [32]
	CHRNA9	Nicotinic receptor and is related to smoking-induced tumor formation [33].

## Data Availability

The data and the codes are available online at https://github.com/bnetlab/DTA_feature_selection (accessed on 15 August 2021).

## Supplementary Materials

The following are available at https://www.mdpi.com/article/10.3390/cancers13174297/s1, Figure S1: Classification accuracy of BRCA for two different cutoffs (a) $z_{t}$ = 2 (blue), (b) $z_{t}$ = 3 (orange). Figure S2: Functional analysis of GBM cluster 1. Figure S3: Functional analysis of GBM cluster 2. Figure S4: Functional analysis of GBM cluster 3. Figure S5: Functional analysis of GBM cluster 4. Figure S6: Enriched biological functions of each subtype of COAD cancer.

## Abbreviations

The following abbreviations are used in this manuscript:

PCA	Principle component analysis
SVD	Singular value decomposition
LDA	Linear discriminant analysis
TCGA	The Cancer Genome Atlas
DE	Differential expression
LASSO	Least absolute shrinkage and selection operator
DTA	Dynamic threshold algorithm
PEEP	Patient-specific perturbation profile
5-CV	Five-fold cross-validation
SVM	Support vector machine
ROC	Receiver operating characteristic
AUC	Area under curve
LUSC	Lung squamous cell carcinoma
LIMMA	Linear models for microarray data
SNF	Similarity network fusion
BRCA	Breast invasive carcinoma
LUAD	Lung adeno-179 carcinoma
PRAD	Prostate adenocarcinoma
COAD	Colon adenocarcinoma
KICH	Kidney chromophobe
GBM	Glioblastoma multiforme
VAR	Maximum variance
