# Supplementary material for: Relevant and Non-Redundant Feature Selection for Cancer Classification and Subtype Detection
Source: Cancers (Basel). 2021 Aug 26;13(17):4297. doi: 10.3390/cancers13174297 (PMC8428340; doi:10.3390/cancers13174297)
Supplement: Supplementary file 1 [file cancers-13-04297-s001.zip › cancers-1361847-supplementary.pdf]

Supplementary Figures

Relevant and Non-redundant Feature Selection for CancerClassification and Subtype Detection

Pratip Rana, Phuc Thai,, Thang Dinh,, and Preetam Ghosh
Department of Computer Science, Virginia Commonwealth University, Richmond, VA 23284, USA

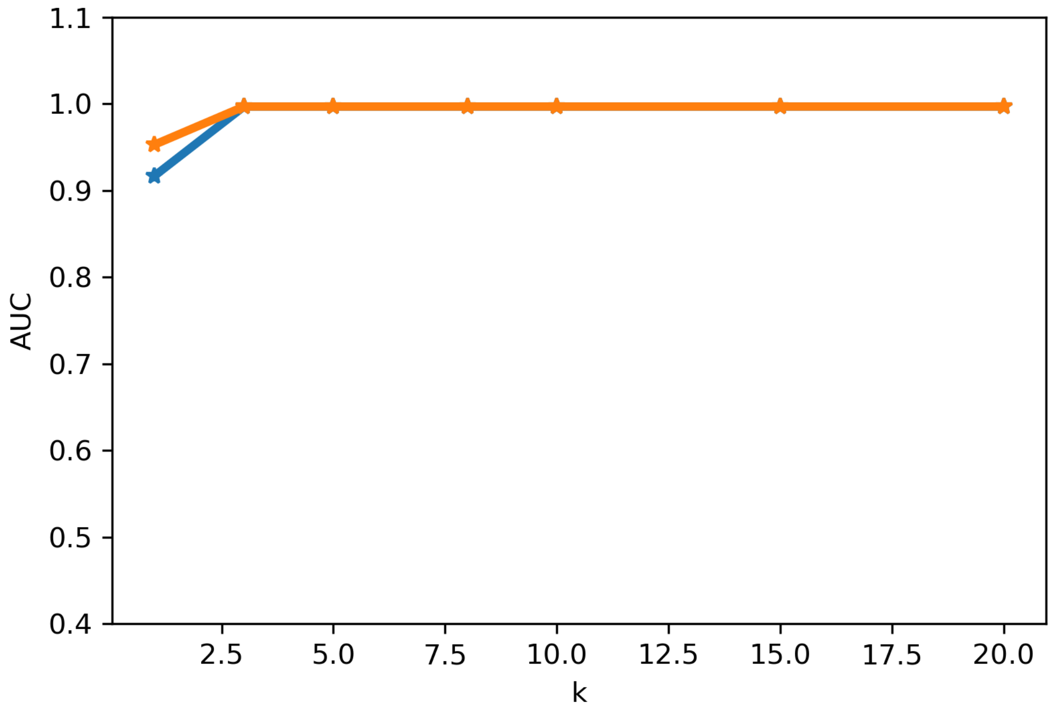

Figure S1: Classification accuracy of BRCA for two different cutoffs a) z_t=2 (blue) b) z_t=3 (orange)

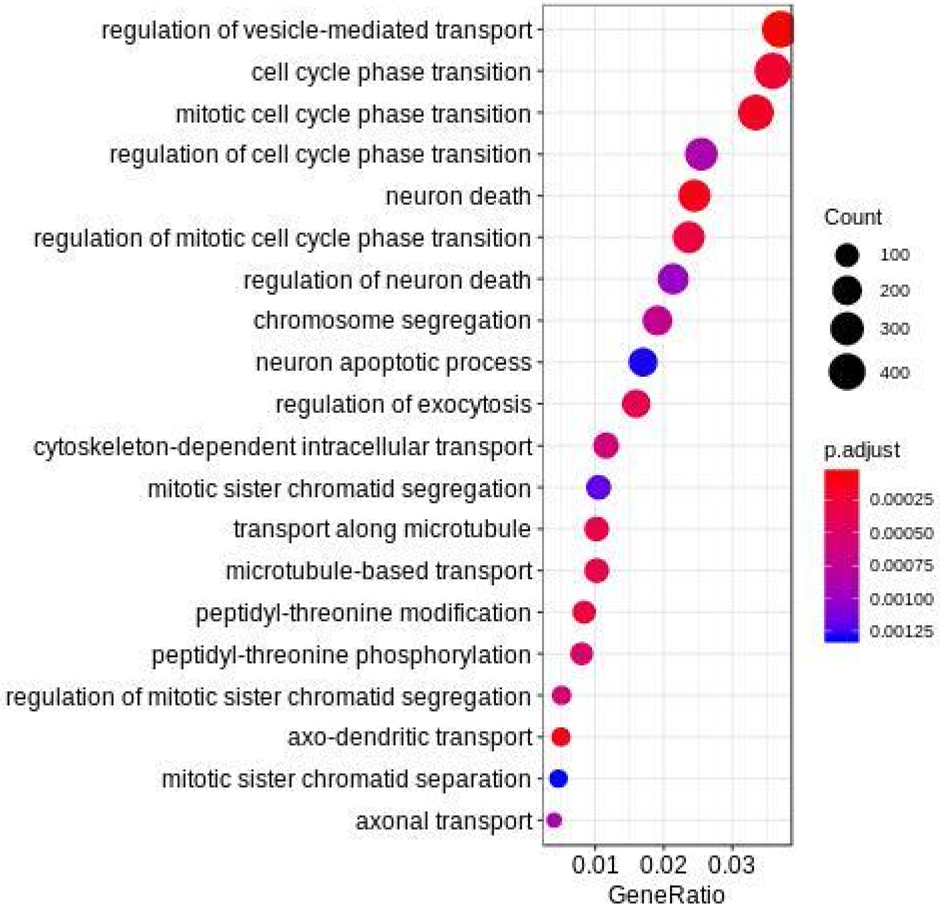

Figure S2: Functional analysis of GBM cluster 1

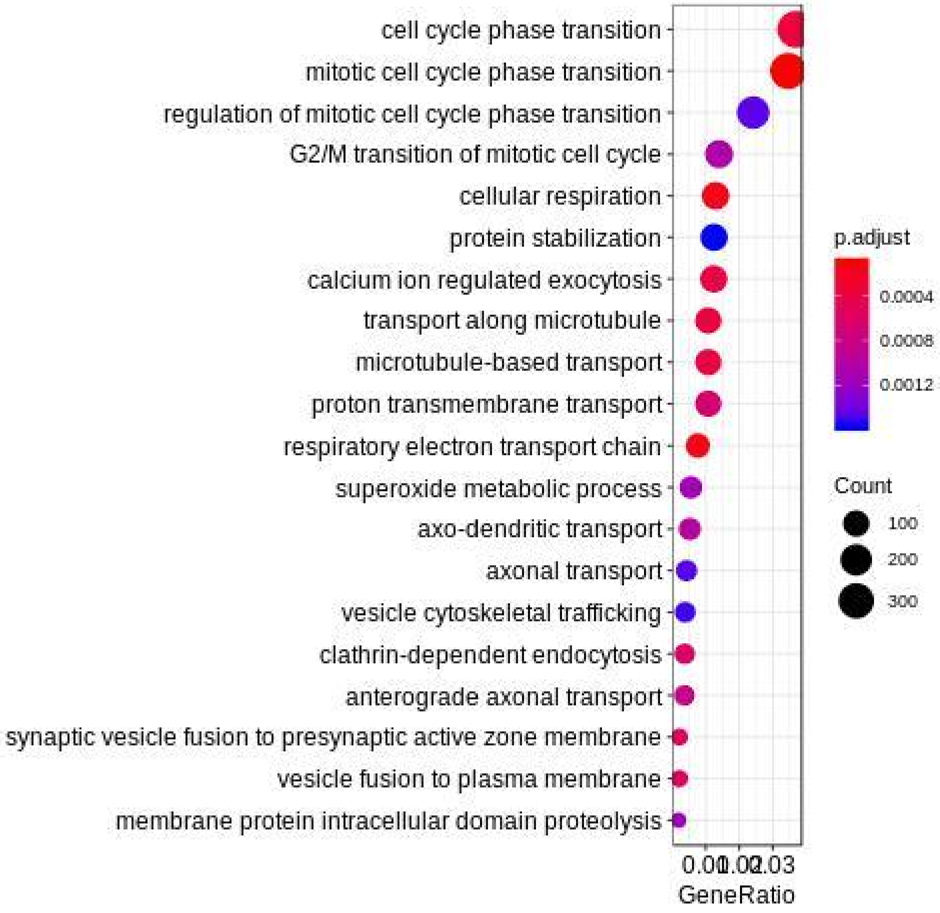

Figure S3: Functional analysis of GBM cluster 2

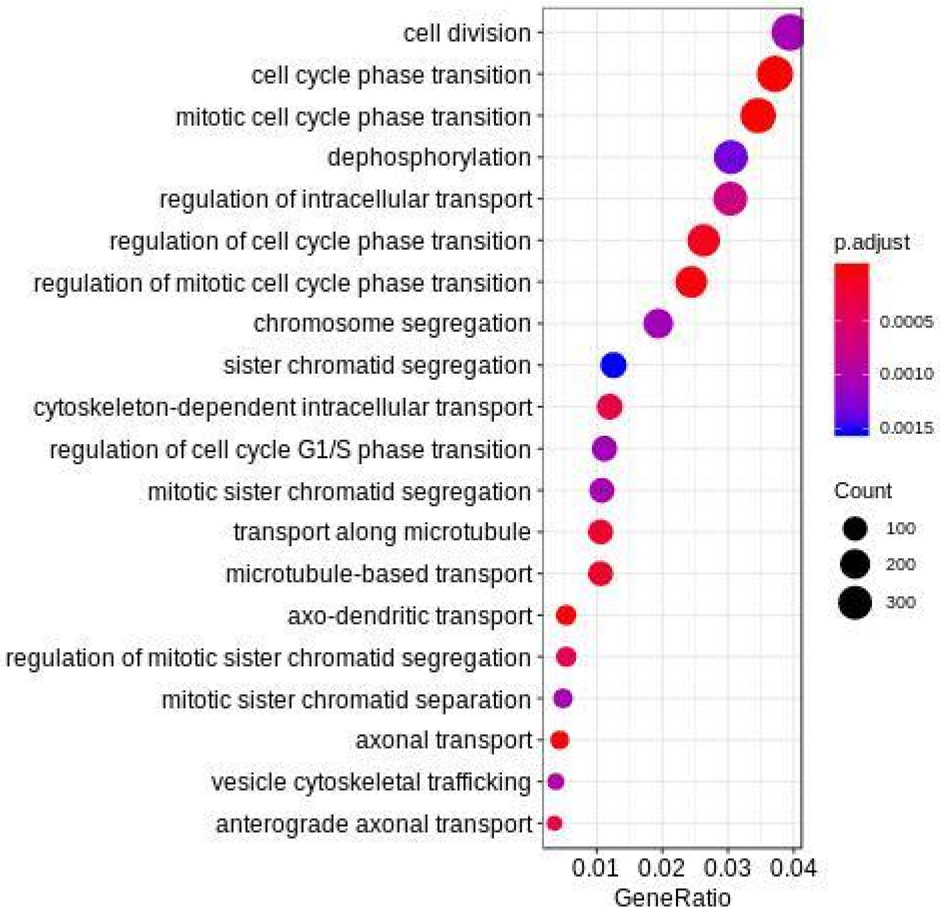

Figure S4: Functional analysis of GBM cluster 3

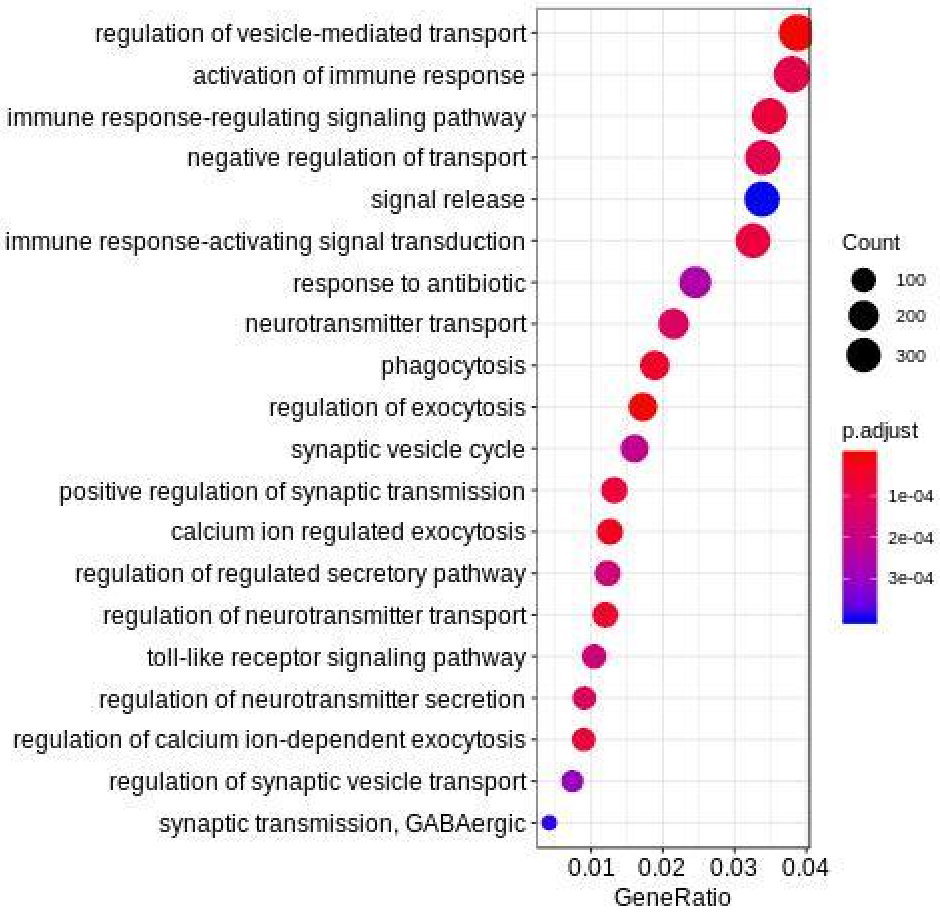

Figure S5 :Functional analysis of GBM cluster 4

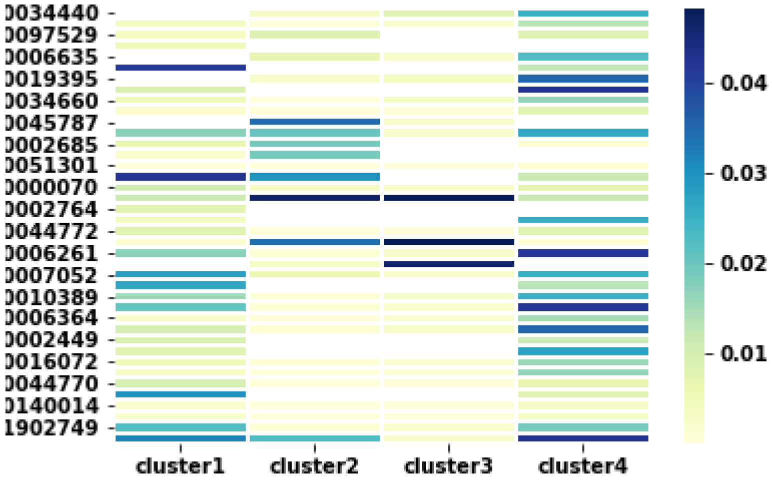

Figure S6: Enriched biological functions of each subtype of COAD cancer
